# DESMOPLASTIC MELANOMA PRESENTING AS PYOGENIC GRANULOMA: REPORT OF A CASE WITH REVIEW OF LITERATURE

**DOI:** 10.4103/0019-5154.70706

**Published:** 2010

**Authors:** B Venkata Ratnam

**Affiliations:** *From the Department of Plastic Surgery, New Medical Centre Specialty Hospital, Abu Dhabi, UAE*

**Keywords:** *Desmoplastic melanoma*, *pyogenic granuloma*, *resemblance*

## Abstract

An elderly female patient was referred to the author for the treatment of a large recurrent pyogenic granuloma in the sole of right foot for a period of 2 years. She underwent excisional surgeries at an outside facility twice in the past. This time, she was treated with wide excision biopsy and the surgical defect was closed with a new technique, the “adjustable suture technique”. Histopathology report confirmed “desmoplastic melanoma” with complete marginal clearance. The wound had healed uneventfully. There were no recurrences at 4-year follow-up.

## Introduction

Conditions without characteristic clinical features, especially if they are rare, can lead to incorrect diagnoses and management. The consequences could be fatal, but might be preventable with a high index of suspicion that follows familiarity with the condition. To the author’s knowledge, desmoplastic melanoma, a rare and disastrous malignancy, presenting as pyogenic granuloma, a common and benign condition, has not been reported yet. This study reports one such case that had misled the treating doctors repeatedly.

## Case Report

This article reports the case of an elderly woman who presented with a painless growth that used to bleed on touch in the sole of her right foot for a period of two years. It was excised twice earlier elsewhere. The details of the earlier presentation, surgery, or histopathology were not available.

**Figure 1:**
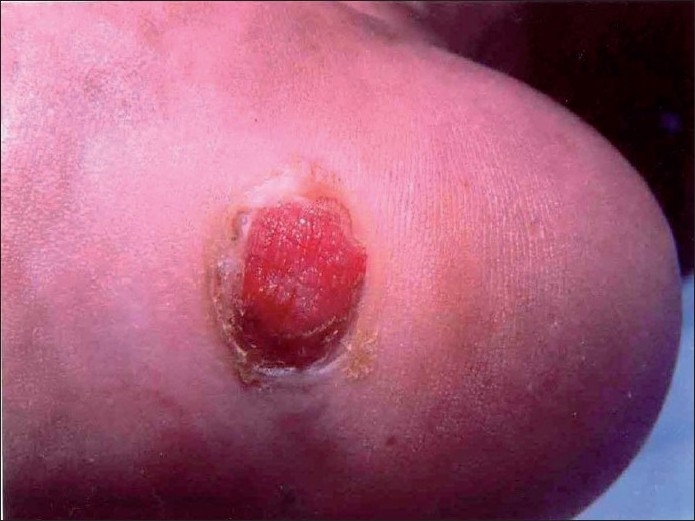
Desmoplastic melanoma appearing as a large pyogenic granuloma

**Figure 2:**
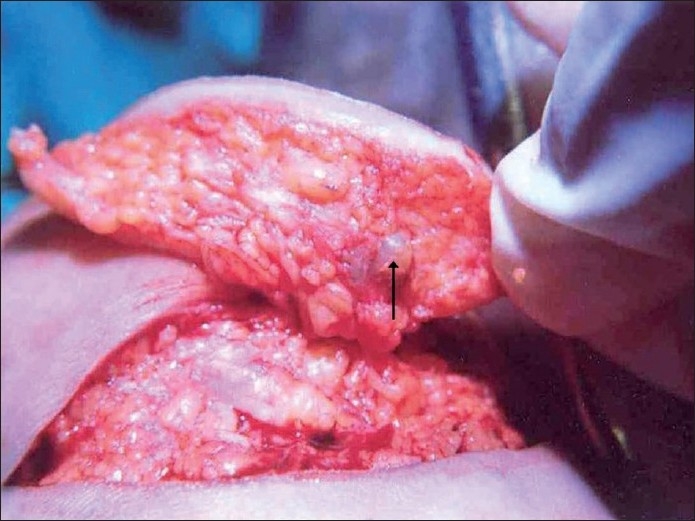
The pigmented soft tissue nodule in the subcutaneous tissue (arrow)

It resembled a large pyogenic granuloma at the junction of instep sole and weight-bearing heel [Figure 1]. There was no similar lesion elsewhere in the body, and there was no evidence of regional or systemic involvement. During a wide excision biopsy procedure, a soft, pigmented nodule of about 1 × 1 cm size was noted in the subcutaneous tissue [Figure 2], which was also included in the specimen. The resultant defect was directly closed in fourteen days’ period using the “adjustable suture technique,” a type of external tissue expansion, initiated by the author.[1]

At four-year follow-up, the wound had healed well and the scar was stable. There was no evidence of local recurrence. Systemic survey and hematological and whole-body CT scanning at four-year follow-up were negative for metastases.

### Histopathology

Separate Hematoxylin and Eosin stained sections from each lesion, the intervening tissue, and the surrounding tissues were examined microscopically.

### The main lesion

**Figure 3:**
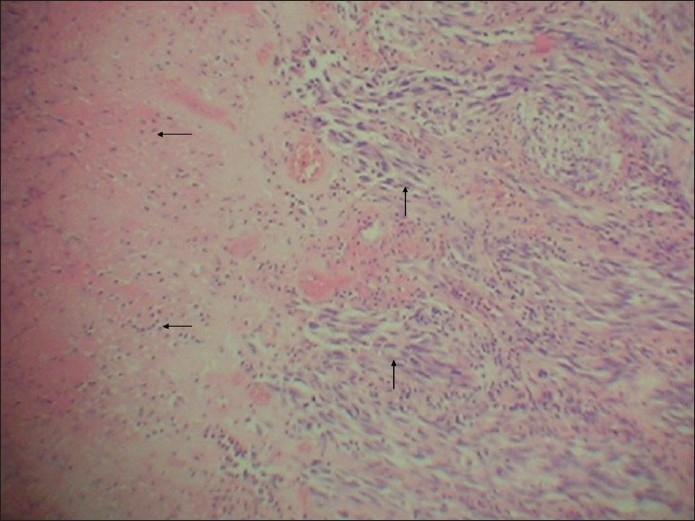
Intradermal location of tumor consisting mainly of spindle cells (vertical arrows) and lymphoplasmacytic infiltration (horizontal arrows) (H and E stain, ×50)

**Figure 4:**
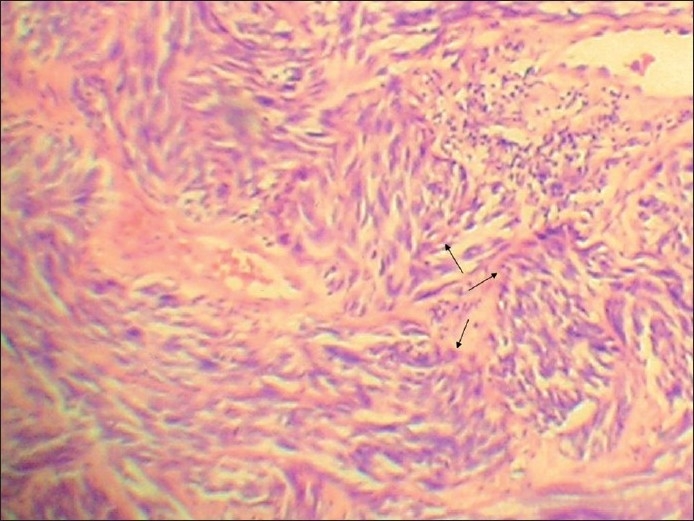
Tumor cells arranged in swirling fascicles reminiscent of DFSP (arrows) (H and E stain, ×100)

**Figure 5:**
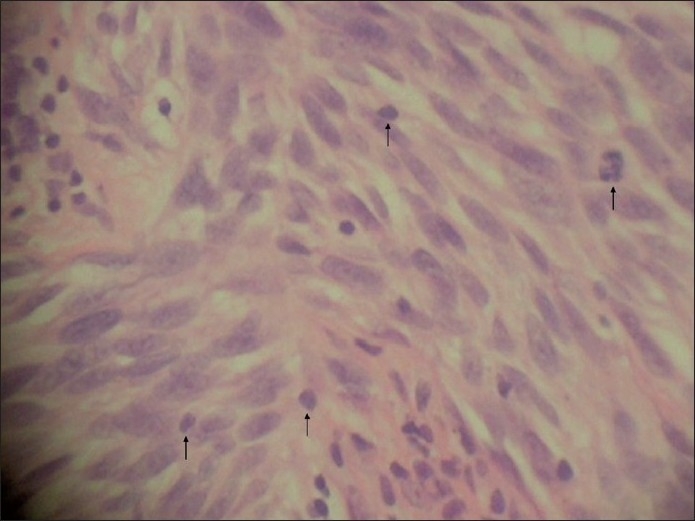
Spindle cells showing intense mitotic activity (arrows) (H and E stain, ×400)

**Figure 6:**
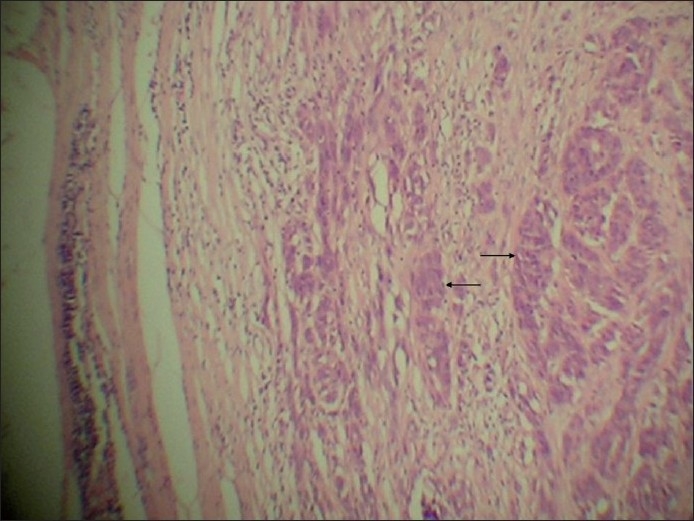
Another area of same tumor showing epithelioid nature of the tumor cells (arrows) (H and E stain, ×100)

**Figure 7:**
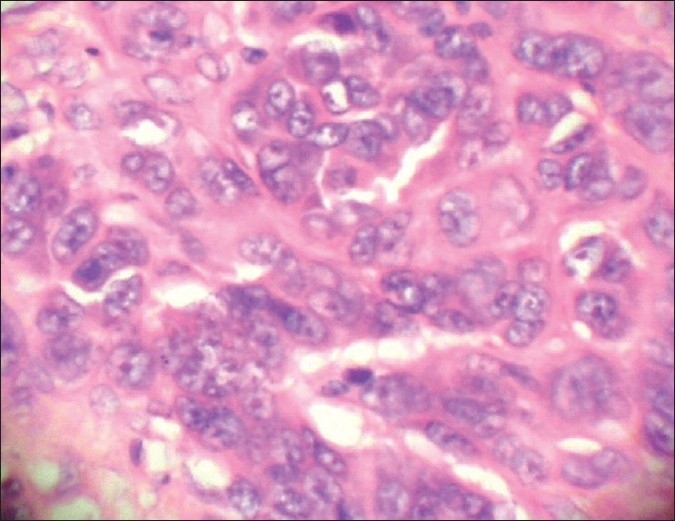
Epithelioid tumor cells with large vesicular nuclei and prominent nucleoli and eosinophilic cytoplasm (H and E stain, ×400)

It showed extensively ulcerated epidermis, covered by necrotic debris and fibrinoid exudates. It had infiltrative and expansile growth patterns in the dermis, extending into the subcutaneous tissue. It mainly consisted of two populations of cells given as follows: (i) The spindle cell population was predominant [Figure 3]. These cells were arranged in swirling fascicles, nests, and bundles exhibiting firoblast-like features. They had elongated, fusiform nuclei and showed mild to moderate degree of pleomorphism. Occasional foci of cells showed perivascular cuffing. In addition, focal storiform-areas, reminiscent of dermatofibrosarcoma protuberans (DFSP) and haemangiopericytoma-like (HPC-like) areas were also found [Figure 4]. The tumor cell mitoses were frequent, being about 30–35/10 high power fields [Figure 5]. (ii) The second cell type was the epithelioid cells [Figures 6 and 7]. They were present in nests and alveolar formations with vesiculated ovoid nuclei [Figure 7]. No cells contained melanin pigment in them. A moderate amount of lymphoplasmacytic infiltrate was present in the stroma [Figure 3]. The lesion was present at Clark’s level V and Breslow’s thickness of 8 mm, with microscopic evidence of all margins of surgical resection appearing free of tumor infiltration.

### The second lesion

**Figure 8:**
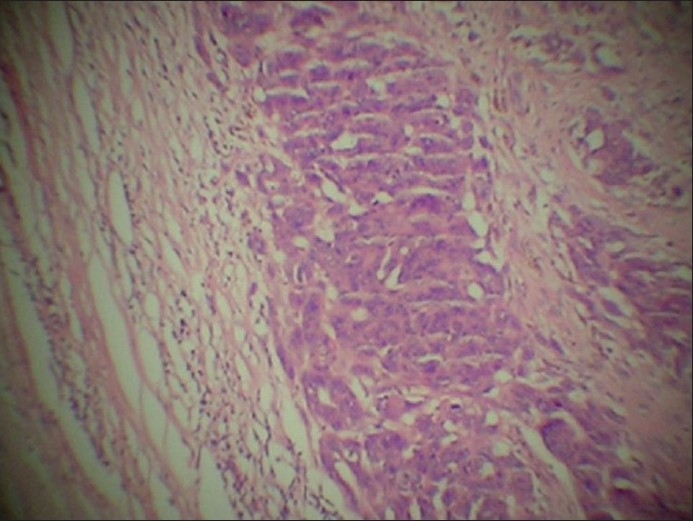
Satellite lesion consisting predominantly of epithelioid tumor cells on the right and fibrous intervening stroma on the left (H and E stain, ×50)

It consisted of small-to moderate-sized cells with small, round nuclei, arranged in alveolar formations and nests [Figure 8]. No spindle cells were seen. A small proportion of these cells showed pale-brown, melanin pigment in their cytoplasm. A moderate amount of lymphoplasmacytic infiltrate was present in its stroma.

### The fibro-fatty subcutaneous tissue between the two lesions

It was free of tumor cells [Figure 8].

### Immunohistochemistry

**Figure 9:**
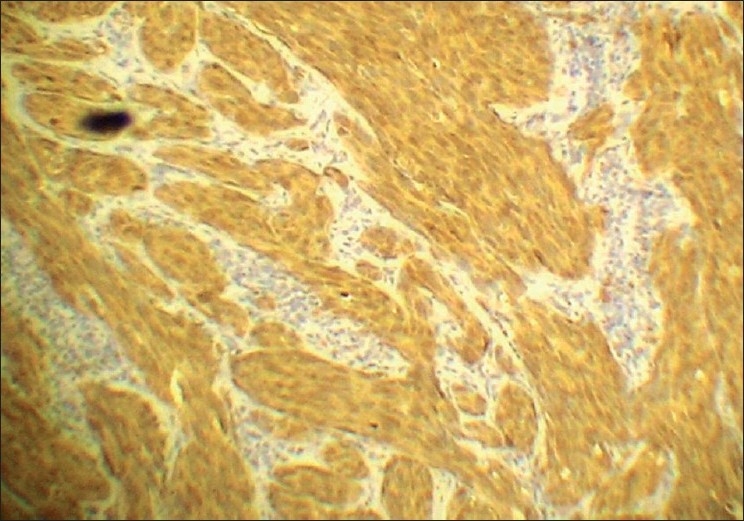
Immunohistochemical staining for S100 – diffuse positivity (Immunoperoxidase, ×400)

**Figure 10:**
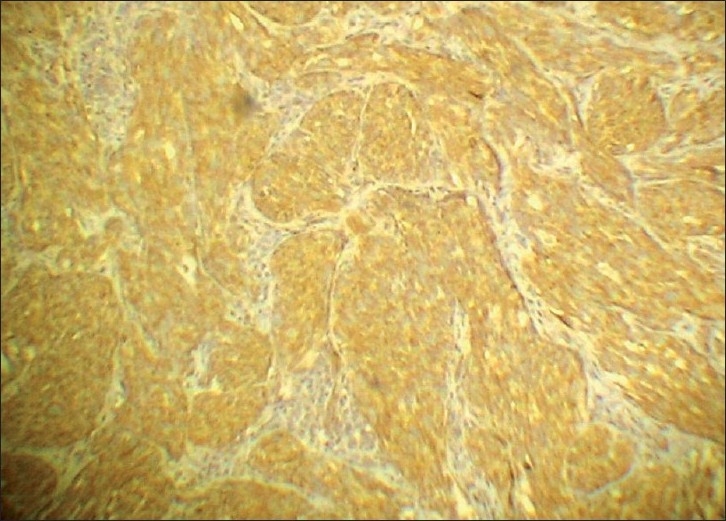
Immunohistochemical staining for Vimentin – strongly positive (Immunoperoxidase, ×100)

**Figure 11:**
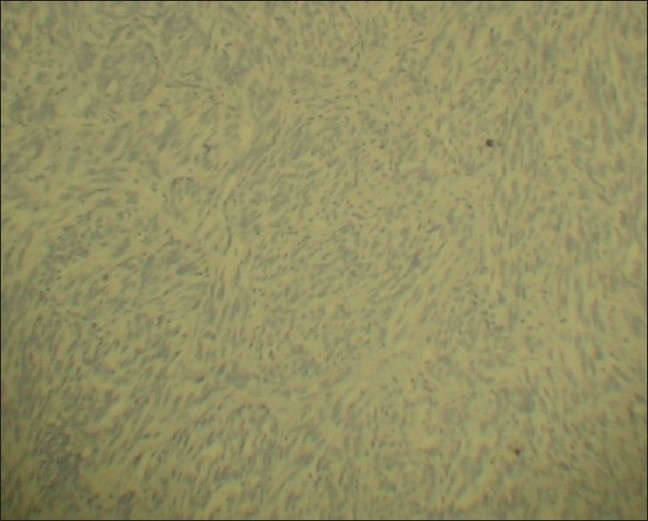
Immunohistochemical staining for Cytokeratin – negative (Immunoperoxidase, ×100)

**Figure 12:**
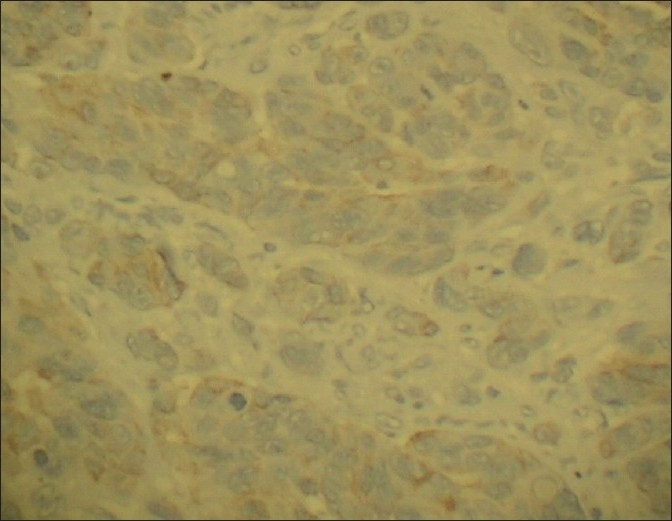
Immunohistochemical staining for HMB 45 – weakly positive (Immunoperoxidase, ×400)

The tumor cells in both the lesions showed diffuse positivity for S-100 [Figure 9] and vimentin [Figure 10], but negativity for cytokeratin [Figure 11]. The cells of the second lesion showed generalized positivity for HMB-45 [Figure 12], but this finding was scanty in the cells of the main lesion.

### Systemic survey

Detailed hematological and whole-body CT scanning both initially and at four-year follow-up were negative for distant metastases.

### Tissue diagnosis

Spindle cell variant of the “combined form” of cutaneous desmoplastic type of amelanocytic melanoma, in vertical growth phase, with marginal clearance and probable satellitism.

### Final diagnosis and staging

Considering the history of repeated attempts at its excision, microscopic finding of scar tissue around the lesions, and the histopathological as well as immunohistochemical features mentioned earlier, the final diagnosis was made as recurrent cutaneous desmoplastic melanoma with macroscopic satellitism, which, as per the new AJCC classification,[2] belongs to: Clinical stage II C (T4b N0 M0) and pathological stage III b (T4 N2C M0).

## Literature Review

Conley *et al*. described “desmoplastic melanoma” in 1971.[3] Subsequently, a vast amount of literature was published about this rare entity. The salient features, in general, of its presentation such as site and clinical appearance, biological behaviour, histopathology and treatment recommendations were found to be common to most, if not all, of the publications, and they are mentioned below.

### Epidemiology

Incidence: 1–2% of all melanomas, De Vita, 2005.[4]
Race: All races, but common in Caucasians.
Sex: Male : Female ratio = 1.75-2 : 1.
Age: Later age group, commonest in the seventh decade of life, Jain S *et al*., 1989.[5]

### Site

It can occur in skin and mucosa of any region of the body. More commonly reported areas of occurrence are as follows:

Males: Sun-exposed areas on the head, neck, and upper part of the trunk.

Females: The extremities.

### Size

Tends to be bulky, many lesions are not recognized until they have reached a substantial thickness.

### Category

Neurectodermal tumor, Carlson JA, 1975[6] /Neural crest tumor, Huttenbach, 2002.[7] Origin: Both from intraepidermal melanocytes as well as de novo, Carlson JA, 1995.[6] Type: Fibrosing variant of spindle cell melanoma, Busam KJ, 2005.[8]

### Biological behaviour

1. Locally aggressive
  - High incidence of local recurrences.
  - Recurrences are attributed to inadequate excision of the primary lesion.
  - History of repeated surgeries with erroneous/mistaken diagnoses.
  - Possibility of aggressive local treatment producing cure.[9]
2. Lymph node metastases: Rare.
3. Distant metastases: Rare. When present, they are common in the lungs.
4. Radiotherapy and Chemotherapy: No proved effectiveness.

### Clinical features

1. Usually nonpigmented lesions.
2. Often mistaken clinically for a scar, a fibroma, a basal cell carcinoma, a fleshy or firm nodule reminiscent of a scar, a sarcoma, or a dermatofibroma.
3. Arise *de novo* or in association with a pre-existing lesion such as lentigo maligna, in which case, a clue to the diagnosis will be available.
4. When de novo, they can present in an unsuspecting manner such as indurated, nonulcerated, and slightly elevated plaque, as a papule, or as a small nodule etc.

Till date, to the author’s knowledge, no report exists describing it presenting as a pyogenic granuloma.

## Histopathology

### Microscopic features

Desmoplasia, Busam, 2005,[8] Markovic *et al*., 2007[10]: Spindle cells (amelanotic, fusiform, melanocytes) dispersed in a prominent collagenous or fibrous tissue stroma. The pure forms contain desmoplasia in more than 90% of the lesion. The combined or mixed forms contain desmoplasia in less than 10% of the lesion, with or without heteromorphism (presence of disparate phenotypes in a single neoplasm). The phenotypic elements include: epithelioid cells, Murali *et al*., 2008,[11] macrophages, smooth muscle, fibroblast-like cells, multinucleated cells, heterotopic bone, lentigo maligna epidermal component, nerve filaments, perivascular lymphoproliferation and focal lymphocytic aggregates.

### Other histological features

They include ulceration, neurotropism, neuroma-like features, cytological atypia, atypical nuclei, high mitotic rate, higher mitotic index, stromal myxoid change, sarcoma-like features, individual necrotic cells, areas of myxoid or storiform appearance, infiltration of the adventitia of the blood vessels, perineurium, endoneurium or local nerves, vertical growth phase while arising in a pre-existing lesion such as a lentigo maligna, common depth of Breslow’s measurement of more than 4 mm, and satellite lesions that are usually microscopic.

### Immunohistochemistry

Diffuse positivity for S-100 and Vimentin, weak positivity or negativity for HMB 45, and negativity for Cytokeratin.

### Diagnosis

Clinical presentation of “desmoplastic melanoma” is a diagnostic trap for the unwary, Mc Carthy *et al*., 2004.[12] The clinical and histological features of this lesion may give rise to perplexing diagnostic and therapeutic dilemmas. A high index of suspicion is warranted for its early and proper diagnosis.

### Prognosis

Conventionally, the prognosis is considered poor, but it is a curable disease with aggressive local treatment alone, Arora *et al*., 2005.[13]

## Treatment

### Wide excision

It alone may be sufficient. To be effective, all the excised specimens must be biopsied, and marginal clearance must be ensured in all the excised specimens. In the absence of marginal clearance, excisional surgery must be repeated until marginal clearance is ensured. Inadequate local treatment leads to local recurrence. Repeated local recurrences can lead to increased incidence of distant metastases. Death is usually due to distant metastases.

### Sentinel lymph node biopsy

It is not usually helpful.

### Follow-up

A careful, long-term follow-up is mandatory.

## Discussion

In the reported case, the nature of initial presentation to dermatologists and operating surgeons of the outside facility was unknown. It had recurred twice after the earlier surgeries. The simple precaution of ensuring the histological “marginal clearance” after the current surgical excision seemed to have made the difference. The local and systemic surveys that were negative at four-year follow-up suggest that, as per Lens *et al*., the condition can be considered as “cured”.[14]

The “adjustable sutures”[1] are mattress sutures applied between the wound edges. Soft materials such as sterile dental rolls are used as bolsters. They are inserted between suture material and skin on either side of the wound, and are retained in place with a slipknot. Loosening of the slipknot, changing of the sterile dental rolls, gentle tightening of the sutures within the pain-tolerance-limit of the patient, and reapplication of the slipknot daily or on alternate days, gradually pulls the wounds’ edges. With time, the wound edges meet each other, permitting direct closure of the wounds and avoiding the need for plastic surgical procedures such as skin grafting, flaps and microsurgery.

## Conclusion

In most cases, a DM is diagnosed only in established long-standing and thick melanomas. Therefore, dermatologists and dermatopathologists should be more aware of this clinicopathologic variant of cutaneous malignant melanoma, de Almeida *et al*., 2008.[15] Accordingly, this case was reported to increase familiarity of treating physicians with this misleading and disastrous malignancy presenting in an innocent manner; and to introduce a simple technique to close the “difficult-to-close” excisional defects that can be used by surgeons of all specialties and capacities.
